# The *PDB_REDO* server for macromolecular structure model optimization

**DOI:** 10.1107/S2052252514009324

**Published:** 2014-05-30

**Authors:** Robbie P. Joosten, Fei Long, Garib N. Murshudov, Anastassis Perrakis

**Affiliations:** a Division of Biochemistry, Netherlands Cancer Institute, Plesmanlaan 121, 1066 CX Amsterdam, The Netherlands; b Structural Studies Division, MRC Laboratory of Molecular Biology, Francis Crick Avenue, Cambridge CB2 0HQ, England

**Keywords:** *PDB_REDO*, validation, model optimization

## Abstract

The refinement and validation of a crystallographic structure model is the last step before the coordinates and the associated data are submitted to the Protein Data Bank (PDB). The success of the refinement procedure is typically assessed by validating the models against geometrical criteria and the diffraction data, and is an important step in ensuring the quality of the PDB public archive [Read *et al.* (2011 ▶), *Structure*, **19**, 1395–1412]. The *PDB_REDO* procedure aims for ‘constructive validation’, aspiring to consistent and optimal refinement parameterization and pro-active model rebuilding, not only correcting errors but striving for optimal interpretation of the electron density. A web server for *PDB_REDO* has been implemented, allowing thorough, consistent and fully automated optimization of the refinement procedure in *REFMAC* and partial model rebuilding. The goal of the web server is to help practicing crystallo­graphers to improve their model prior to submission to the PDB. For this, additional steps were implemented in the *PDB_REDO* pipeline, both in the refinement procedure, *e.g.* testing of resolution limits and *k*-fold cross-validation for small test sets, and as new validation criteria, *e.g.* the density-fit metrics implemented in *EDSTATS* and ligand validation as implemented in *YASARA*. Innovative ways to present the refinement and validation results to the user are also described, which together with auto-generated *Coot* scripts can guide users to subsequent model inspection and improvement. It is demonstrated that using the server can lead to substantial improvement of structure models before they are submitted to the PDB.

## Abstract 2

The *PDB_REDO* pipeline aims to improve macromolecular structures by optimizing the crystallographic refinement parameters and performing partial model building. Here, algorithms are presented that allowed a web-server implementation of *PDB_REDO*, and the first user results are discussed.

## 1. Introduction   

Crystallographic structure elucidation is a stepwise process with many decision points, and is therefore complex and labour-intensive. Over the years, this process has become more and more streamlined by automation. The crystallo­graphic process, starting from the diffraction experiment itself, has greatly benefitted from faster computers and advances in crystallographic software. Automated pipelines are available for data reduction (*e.g.* Otwinowski & Minor, 1997 ▶; Vonrhein *et al.*, 2011 ▶; Krug *et al.*, 2012 ▶; Monaco *et al.*, 2013 ▶; Winter *et al.*, 2013 ▶), experimental phasing (*e.g.* Panjikar *et al.*, 2005 ▶; Terwilliger *et al.*, 2009 ▶; Pannu *et al.*, 2011 ▶), molecular replace­ment (*e.g.* Keegan & Winn, 2007 ▶; Long *et al.*, 2008 ▶; McCoy *et al.*, 2007 ▶), density-map tracing and model building (*e.g.* Perrakis *et al.*, 1999 ▶; Ioerger & Sacchettini, 2002 ▶; Cowtan, 2006 ▶; Terwilliger *et al.*, 2008 ▶) and combinations thereof (*e.g.* Brunzelle *et al.*, 2003 ▶; Holton & Alber, 2004 ▶; Kroemer *et al.*, 2004 ▶).

The *PDB_REDO* pipeline (Joosten *et al.*, 2012 ▶) focuses on automating the final steps of the crystallographic process, *i.e.* the optimization of the structure model through refinement and rebuilding. We have called this procedure ‘constructive validation’ because throughout the process model quality assessment is used to determine whether the structure model can be improved. This takes away the need to define what is ‘good’ or ‘bad’, which is difficult for many metrics of model quality and can let ‘good enough’ stand in the way of ‘excellent’. For example, an *R*_free_ value of 18% is certainly ‘good’ under most circumstances, but if it could be lowered to 16% this would be preferred. Automation allows a comprehensive approach to exploiting a large number of possible ways that can lead to a more accurate and reliable model, while minimizing user intervention. In addition, automation provides a consistency that would be difficult to achieve manually. For instance, *PDB_REDO* checks every amino-acid side chain to determine whether an alternative rotameric conformation can be found with an equal or better fit to the electron density. This is a daunting task if performed manually, especially for large structures, but rather trivial to perform computationally in a systematic manner: in many cases, this exhaustive search leads to substantial improvement of the geometric quality of the model (Joosten *et al.*, 2011 ▶). *PDB_REDO* also optimizes refinement parameters, *e.g.* by finding good restraint weights and selecting the most suitable *B*-factor model. This also frequently leads to model-quality improvement, but again is quite time-consuming when performed manually.

The *PDB_REDO* pipeline was developed as command-line-oriented software for Linux. This allows high-throughput analysis of large sets of structure models, which is used to create a data bank of optimized and consistently treated PDB entries (Joosten *et al.*, 2009 ▶). Our recent developments of *PDB_REDO* have made it an attractive tool for helping model refinement prior to submission to the PDB, to help ongoing crystallographic studies. However, the rather abstract textual output and a number of third-party software dependencies make the installation and routine/efficient use of *PDB_REDO* challenging. Moreover, as *PDB_REDO* is a work-in-progress project, we wanted to always make the latest software available to end users. To alleviate these barriers for end users, we chose to implement a *PDB_REDO* web server (http://xtal.nki.nl/PDB_REDO). Additions to the *PDB_REDO* pipeline as well as new visual output that makes *PDB_REDO* more accessible to end users are discussed.

## 2. Specific amendments to the *PDB_REDO* pipeline   

The *PDB_REDO* procedure consists of model refinement in *REFMAC* (Murshudov *et al.*, 1997 ▶, 2011 ▶), rebuilding by *Centrifuge* (which deletes waters without electron density), *SideAide* [which (re)builds side chains in rotameric conformations] and *pepflip* (which flips the orientation of peptide planes to improve the fit with electron-density maps and the Ramachandran plot; Ramachandran *et al.*, 1963 ▶) (Joosten *et al.*, 2011 ▶), and validation in *WHAT_CHECK* (Hooft *et al.*, 1996 ▶). Additional validation is performed by *pdb-care* for carbohydrates (Lütteke & von der Lieth, 2004 ▶) and *FoldX* (Guerois *et al.*, 2002 ▶) for calculation of the Gibbs folding energy. These programs are linked together by a large set of decision-making algorithms which we have discussed in detail previously (Joosten *et al.*, 2012 ▶).

Many decision-making algorithms in *PDB_REDO* aim to optimize the refinement parameterization for *REFMAC*. Two new features were added to improve this parameterization.

(i) If the provided reflection data extend to a resolution higher than that used previously to refine the model (*e.g.* the data extend to 2.0 Å resolution, whereas the header of the PDB file shows that it was refined with data up to only 2.5 Å resolution), paired refinement (Karplus & Diederichs, 2012 ▶) is used to establish a new resolution cutoff. In this procedure, the resolution is extended step by step. In each step two models, one at a lower and one at a higher resolution, are refined and the fit against only the data for the lower resolution cutoff is calculated. If the model that was refined with the higher resolution data fits the data in the common resolution range equally well or better, then the new, higher, resolution cutoff is accepted.Notable differences between the protocol described by Karplus and Diederichs and the *PDB_REDO* implementation are that (1) the steps have an equal size in the number of observed reflections (as established by the *PDB_REDO* program *binliner*) rather than an equal size in resolution steps, ensuring that the effective information content of each subsequent step can be predicted to be lower (owing to increasing noise in the diffraction data) than that of the previous step, and (2) not only *R*_free_ but also the weighted *R*_free_, the free correlation coefficient and the free log likelihood are used to test the fit to the reflection data. Here, the *PDB_REDO* program *resolute* rejects the higher resolution model if two or more of these metrics show (numeric) deterioration.
(ii) Some input models have occupancies for ligands and nonstandard amino acids or bases (residues) that are unlikely. Typically, these are residues with some atoms marked as ‘not there’ by occupancies set to 0.00 or 0.01 or that have at least three different occupancies among their atoms. Such models are likely to represent an artificial way to improve the fit to the data rather than any chemical reality. For this reason, *PDB_REDO* defines a single occupancy per residue that is refined by *REFMAC*.

An additional improvement concerns the calculation of *R*_free_ (Brünger, 1992 ▶) in cases where the test set of reflections used for calculation of *R*_free_ is very small and stochastic effects can cause *R*_free_ for a particular set of test reflections to be substantially (up to several percentage points) higher or lower than for an alternative test-set selection. In such cases, a single value of *R*_free_ can be misleading and *k*-fold cross-validation (where *k* is the number of alternative test sets, *e.g.* 20 if the original test set constitutes 5% of all reflections) can be used to obtain averages and standard deviations for *R*, *R*_free_ and their difference. In *PDB_REDO*, *k*-fold cross-validation (Picard & Cook, 1984 ▶; Kleywegt & Brünger, 1996 ▶) is used if the test set is smaller than 500 reflections. To ensure that the alternative test sets are ‘free’, the input model for the cross-validation is perturbed by resetting the atomic *B* factors to a fixed value or by small shifts to the atomic coordinates in cases where individual atomic *B* factors cannot be used.

Two new validation routines were added to *PDB_REDO*.

(i) Real-space *R* factors and correlation coefficients, RSR (Jones *et al.*, 1991 ▶) and RSCC (Branden & Jones, 1990 ▶), respectively, are calculated by the program *EDSTATS* (Tickle, 2012 ▶).
(ii) Ligands and the interactions with their binding sites are validated with *YASARA* (Krieger *et al.*, 2002 ▶). *PDB_REDO* currently reports the ligand’s heat of formation (as a measure of conformational strain), the number of atomic clashes, the number of hydrogen bonds and their total energy, and the number of hydrophobic, π–π and cation–π interactions and their total magnitude compared with knowledge-based potentials (Krieger *et al.*, 2009 ▶) for the original and the final structure model. Although none of these values can show the absolute quality of the model, owing to the lack of a concrete target value, the changes in these values can help to select which model is more suited for follow-up studies of the ligand-binding interactions (Cereto-Massagué *et al.*, 2013 ▶).

## 3. Implementing model quality metrics   

### 3.1. Defining significance thresholds for changes in indicators of global model quality   

The optimization of a structure model leads to changes at the global and at the local level, which can be quantified by the changes in the values of specific metrics that assess the fit of the model to the experimental data or the conformity of atomic geometry to *a priori* chemical knowledge. An important question is how to define the significance thresholds in the changes of established metrics. Such thresholds are important for the subsequent visualization of the results.

For *R*_free_, we estimate 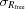 to be (Kleywegt & Brünger, 1996 ▶)
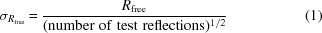
and we subsequently define a change of 2.6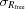 as significant (as it corresponds to a *p*-value of 0.01 assuming a normal distribution).

Similarly, for the change of the free correlation coefficient a *Z*_change_ score of 2.6 is required. This *Z*_change_ score is calculated as
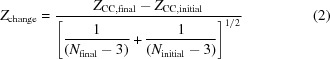
where *N* is the number of data points used to calculate the correlation coefficient and where the *Z*_CC_ values are obtained by transforming the correlation coefficients of the initial and the final model using the Fisher transformation (Fisher, 1915 ▶), 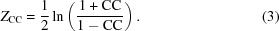
There are no well defined targets for bond-length and bond-angle deviations expressed as root-mean-square *Z*-scores (r.m.s.*Z*), other than that the scores should not exceed 1.0 (Tickle, 2007 ▶). Therefore, an alternative scheme to mark changes that are likely to be significant was devised: if the initial r.m.s.*Z* is greater than 1.0 then any increase is considered to be a significant deterioration and any decrease to be a significant improvement. In addition, an increase from an r.m.s.*Z* value of less than 1.0 to an r.m.s.*Z* values of greater than 1.0 is always considered to be a significant deterioration. All other changes are considered to be insignificant.

**Figure 1:**
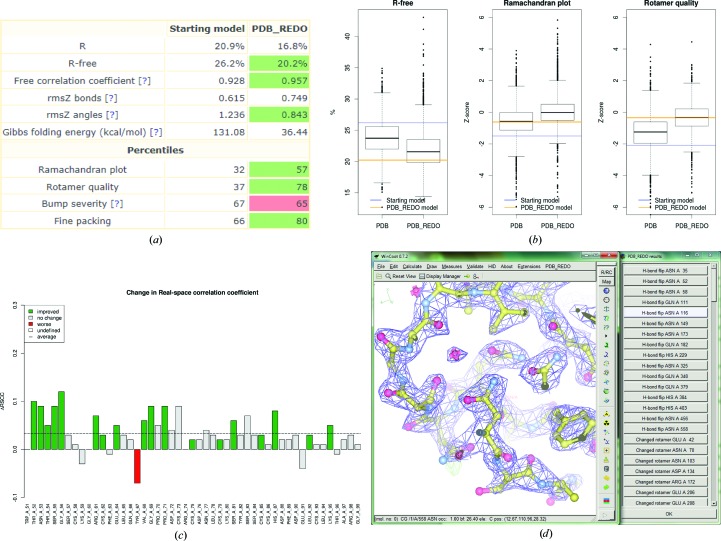
Example output of the *PDB_REDO* web server. (*a*) The table of global structure model quality indicators for the starting model and the final *PDB_REDO* model. Significant improvements are marked in green and deteriorations in red. See the main text for a description of the significance levels. The blue question marks are hyperlinks to the *PDB_REDO* FAQ. (*b*) Box-and-whisker plots of global model quality indicators. The plots represent quality scores of at least 1000 structure models (from the PDB or their PDB_REDO data bank counterparts) that have a resolution close to that of the user’s model. The whiskers extend to 1.5 times the inter-quartile range. The quality of the starting model is indicated as a blue line and that of the final model as an orange line. (*c*) Changes in real-space correlation coefficient per residue (for the N-terminal part of a study case). Significant improvements are coloured green and deteriorations red, while grey denotes no significant change and white denotes undefined significance; the dotted line denotes the average change across the whole protein. This plot also demonstrates that the significance of a change depends on the magnitude but also on the size of the residue and the resolution of the diffraction data. (*d*) A *Coot* (Emsley *et al.*, 2010 ▶) window with a button list that highlights the structural changes made by the *PDB_REDO* pipeline.

Overall geometric quality scores are presented as the percentile rank with respect to all PDB entries (Bernstein *et al.*, 1977 ▶; Berman *et al.*, 2007 ▶) that are also represented in the PDB_REDO data bank (Joosten & Vriend, 2007 ▶). Given that about 70 000 structures are available in the PDB_REDO data bank, a change in percentile rank of a single point is thus equivalent to overtaking some 700 PDB entries for that specific metric. Thus, a change in percentile rank of one or greater is considered to be a significant change. The Ramachandran-plot, rotamer-quality and fine-packing percentiles are directly derived from the corresponding *Z*-scores from *WHAT_CHECK* (Vriend & Sander, 1993 ▶; Chinea *et al.*, 1995 ▶; Hooft *et al.*, 1997 ▶); the bump-severity percentile is based on the weighted bump severity BS_w_ (4),[Disp-formula fd4] which penalizes severe atomic clashes [*i.e.* clashes with large van der Waals (VdW) overlaps] and downweights minor clashes. We use this weighted score to downweight the minor clashes that could be brought about by too liberal VdW restraints or refinement without riding H atoms, and focus on bumps that are caused by actual fitting errors,
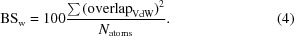
These global model quality scores (*R*, *R*_free_, free correlation coefficient, bond-length r.m.s.*Z*, bond-angle r.m.s.*Z*, Gibbs folding energy, Ramachandran-plot percentile, rotamer-quality percentile, bump-severity percentile and fine-packing percentile) are presented in a tabular form (Fig. 1 ▶ *a*). The significant changes, according the criteria outlined above, are marked in red for deteriorations and green for improvements.

### 3.2. Showing changes in global model quality   

Here, we aim to show graphically how key quality indicators of the model at hand compare with similar resolution structures in the PDB and the PDB_REDO data bank before and after applying the *PDB_REDO* pipeline. To create this graph, we first retrieve ‘on the fly’ the structures that are closest in terms of resolution to the structure at hand. For this, we sort all available entries by their distance in resolution space from the working model. The top 1000 nearest neighbours are then chosen, including any structures that have exactly the same distance as the 1000th nearest neighbour. Three global quality metrics (*R*_free_, Ramachandran-plot quality and rotamer quality) for all of these structures are used to create box-and-whisker plots representing the distributions of these values from these at least 1000 structure models of similar resolution (Fig. 1 ▶ *b*). Outliers are flagged according to the 1.5 inter-quartile range (IQR) criterion (Tukey, 1977 ▶). These underlying distributions are calculated and plotted for both the PDB and the PDB_REDO data bank, and the values for the model at hand are then plotted as a blue line (before *PDB_REDO*) and an orange line (after *PDB_REDO*), allowing the user to judge how the structure models before and after *PDB_REDO* compare with similar published structure models and with the same structure models as optimized in the PDB_REDO data bank.

### 3.3. Showing local model changes   

To present a static view of local changes in the model, per-residue changes in the fit to the electron-density map are plotted as the change in real-space correlation coefficient (RSCC) as calculated by *EDSTATS* (Fig. 1 ▶ *c*). If the RSCC of a residue has an absolute *Z*_change_ of >2.6, as calculated in (2), it is marked in red for deterioration and green for improvement; if not, it is marked in grey. As *N*_initial_ and *N*_final_ in (2) now represent the number of independent map grid points used to calculate the RSCC, *Z*_change_ not only depends on the magnitude of the RSCC change but also on the size of the residue and the resolution of the electron-density map. This means that at lower resolution *Z*_change_ becomes undefined for small compounds such as waters or ions. These residues are marked in white.

In addition to the static visuals implemented in the web browser, we want to enable the user to have an easily interactive view: local changes in the conformation of the structure such as changed rotamers, flipped peptides, flipped His/Asn/Gln residues, side chains, deleted waters or completed side chains are listed in scripts (in Scheme and Python format) that can be read directly in *Coot* (Emsley & Cowtan, 2004 ▶; Emsley *et al.*, 2010 ▶). The scripts create a pop-up *Coot* window with a list of buttons that guide the user through all of the structural changes (Fig. 1 ▶ *d*). The scripts are also available for the PDB_REDO data bank and are automatically loaded when the current version of the *PDB_REDO* plugin for *Coot* (Cereto-Massagué *et al.*, 2013 ▶) is used.

## 4. Server implementation   

The server is based on the framework of the YSBL software server (Long *et al.*, 2008 ▶) consisting of a front-end web server for user interaction and a back-end computation node that manages the* PDB_REDO* jobs. The computation node (with four six-core Intel Xeon E5-4610 processors and 64 GB RAM) is set up to run a maximum of 46 *PDB_REDO* jobs in parallel. When this capacity is fully used, additional jobs are queued on a ‘first come, first served’ basis. Job ‘requests’ from the web server are accumulated as single files with references to the job parameters, based on user input. The web server and the computation node simply share common disk space for input, control and output files; the actual jobs are run on a local disk of the computation node. Job management on the computation node (starting and stopping processes) is implemented as a series of *crontab* scripts that are executed every few minutes and collect information from the files created by the web server.

**Figure 2:**
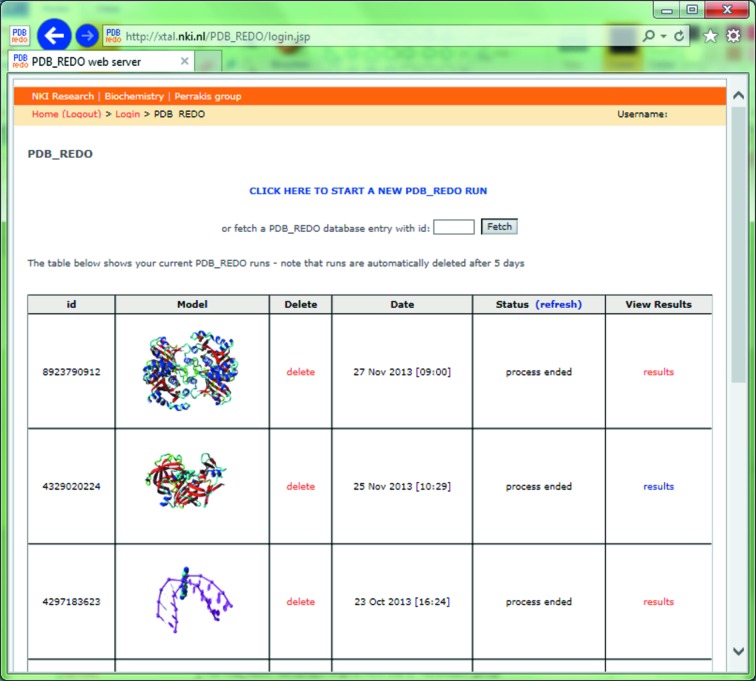
The personal, password-protected, workspace on the *PDB_REDO* web server from which jobs can be submitted, inspected and deleted.

New users register an account, after which immediate access is granted (no approval or control mechanism is in place). Each user has their personal, password-protected, workspace to submit and monitor *PDB_REDO* jobs (Fig. 2 ▶). Submitting a job consists of uploading reflection data in MTZ format (the correct data columns are automatically selected by the program *kollumer*), model coordinates in PDB format and (optionally) a file with geometric restraints describing non­standard compounds and atomic links for *REFMAC*.

Detailed information on job progress is accumulated in real time as the *PDB_REDO* procedure goes through the several steps, and decisions are explained to the user. After a job is completed, the model change indicators described in §[Sec sec3]3 are computed and displayed in tabular and graphical form. A table with hyperlinks to the optimized structure model, an MTZ file with electron-density map coefficients, a keyword file to run *REFMAC* with the settings as optimized by *PDB_REDO*, the *Coot* scripts, a detailed log file and a compressed file with additional validation results, intermediate models and other files is also available.

Job results are stored for up to 30 days, but can be deleted sooner by the user. All data are considered private, except for statistical indicators of the *PDB_REDO* performance. After job deletion, only these statistical data (no coordinates or reflections) are retained.

The *PDB_REDO* server is set up to allow updates without taking the server offline. This way, the server can always run the latest version of the pipeline, allowing us to fix bugs that stop jobs from completing at very short notice. More importantly, new developments in the *PDB_REDO* pipeline are made available to users with minimal delay.

The server is free to use for academic and commercial users as long as they already hold a licence for the *CCP*4 suite (Winn *et al.*, 2011 ▶). License requirements for *YASARA* and *FoldX* have been waived by their respective developers because only small parts of the programs’ functionality are used.

## 5. Analysis of user jobs   

To evaluate the performance of the *PDB_REDO* server, we analysed the results of 1167 jobs from data sets that already contained a test-set selection for *R*_free_ and were submitted from December 2013 to April 2014. It should be noted that the jobs on the server do not constitute a carefully selected ‘test set’.

(i) The jobs are not independent because users can upload different structure models corresponding to the same data set, or a new data set (*e.g.* with different resolution cutoffs) for the same structure model.
(ii) Models might be uploaded after only a round of manual model building, or even directly after molecular-replacement solution (we have observed users testing alternative solutions to see which one shows the best refinement results), or can be very good models submitted for validation or a final polishing round just before submission to the PDB.

**Figure 3:**
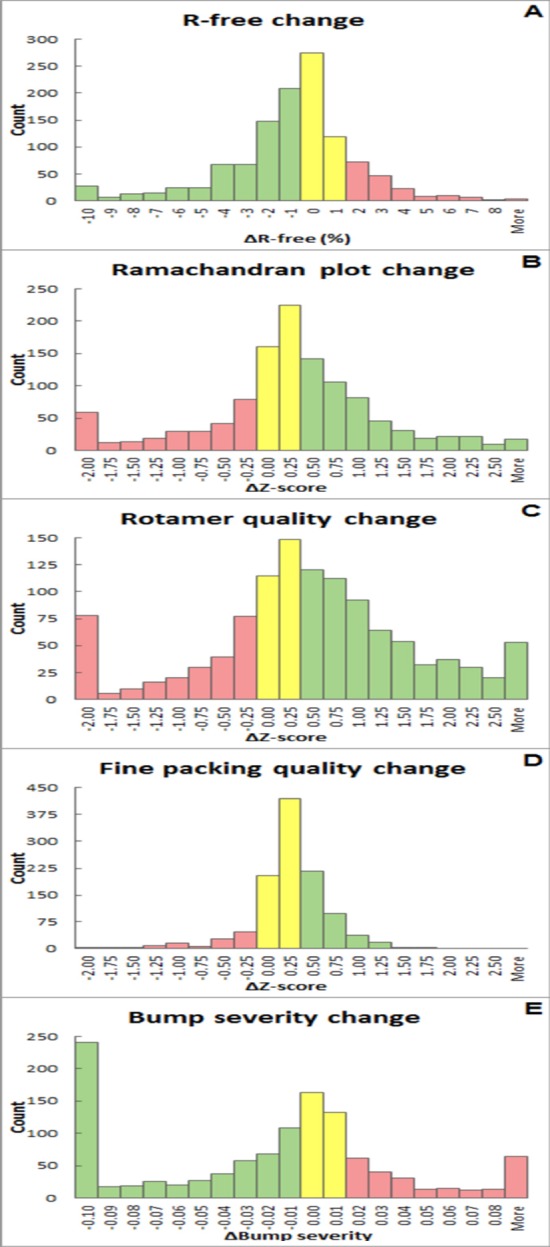
Comparison of model quality scores of starting models and final *PDB_REDO* models as histograms of score change. The values on the *x* axis mark the upper limit of each bin. Improvements are marked in green, deteriorations in red and neutral changes in yellow. (*a*) *R*_free_ as calculated by *REFMAC* (Murshudov *et al.*, 2011 ▶). (*b*) Ramachandran plot *Z*-score from *WHAT_CHECK* (Hooft *et al.*, 1996 ▶). (*c*) Rotamer normality *Z*-score from *WHAT_CHECK*. (*d*) Fine (second-generation) packing *Z*-score from *WHAT_CHECK*. (*e*) Weighted bump severity score (see equation 4).

Fig. 3 ▶ shows the distributions of model quality changes resulting from using *PDB_REDO* in terms of *R*_free_ and four geometric quality metrics. *R*_free_ improvements of several percentage points are observed, with some of greater than 10 percentage points. A total of 40% of the runs result in an *R*_free_ improvement of greater than 2.6 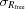. Deteriorations of *R*_free_ of the same magnitude occur for 9% of the runs: however, more than a quarter of these cases had initial *R*_free_ values that were lower than the initial *R*, indicating that the test set was not ‘free’ (*i.e.* the data in the test set were previously used to construct the structure model; see §2.2.1 of Joosten *et al.*, 2012 ▶) at the start of the *PDB_REDO* run and that *R*_free_ is underestimated. In such cases *R*_free_ can increase by several percentage points when the refinement converges. We also, rather expectedly, observe that the chance of improving *R*_free_ is higher if the initial *R* is high: at very low initial *R* factors (lower than 15%) there is little room for improvement and only 22% of all cases shows a significant drop in *R*_free_; this success rate increases to 76% for cases with an initial *R* factor of between 35 and 45%. At even higher starting *R* factors the success rate drops again to 52%: this may be explained by the presence of models that either need substantial rebuilding or models that are incorrect molecular-replacement solutions of a trial subset.

Looking at the basic global model quality scores we described above, we see reassuring trends.

(i) Ramachandran-plot *Z*-score improvements of greater than 0.25 occur for 42% of the runs, while similar deteriorations only occur 24% of the time. Inspecting the 35 best-performing and the 35 worst-performing runs in terms of Ramachandran-plot quality, *i.e.* 3% on each side of the distribution, shows that the fit of the input structure model to the experimental data is very important. Only two of the best cases had starting *R* factors over 40%, whereas 21 of the worst cases had *R* values higher than 40%. That indicates that *PDB_REDO* is more likely to improve models that are already in the later stages of model building (as it was designed to do) rather than early models originating, for example, from molecular replacement. However, we are positively surprised that even early models can in some cases be improved by *PDB_REDO*, and we consider this to be an appropriate use of the server.
(ii) Substantial improvement in side-chain rotamer quality is observed for more than half (53%) of all *PDB_REDO* runs; this is expected, but also reassuring, as the *SideAide* program in the *PDB_REDO* pipeline explicitly optimizes rotamers, albeit using a different rotamer set than that used for validation. Very large deteriorations in rotamer quality are observed almost exclusively in cases that have very high starting *R* factors: such models typically have very poor corresponding electron-density maps that do not allow optimal placement of rotamers.
(iii) Changes in fine-packing quality are much smaller and very few extreme changes are encountered. Improvements (34%) outweigh deteriorations (10%) by three to one.
(iv) More than half of the structure models improve in terms of weighted bump severity (53%), whereas 22% deteriorate. It should be noted that in terms of bump severity the models discussed here are much worse than what is common in the PDB: the average weighted bump severity of the test set corresponds to the first percentile for the input models. This percentile improves to 22% for the average of models produced by the *PDB_REDO* server. It is perhaps not surprising that the largest improvements are found in correct molecular-replacement solutions that were submitted to *PDB_REDO* without any prior refinement.

## 6. Outlook   

*PDB_REDO* is under active development, which means that new features will be added to improve the results. The results show that particularly models that do not (yet) fit the experimental data well are likely to benefit from special treatment. For instance, more systematic use of jelly-body refinement in *REFMAC* (Murshudov *et al.*, 2011 ▶) may improve the performance of *PDB_REDO*, especially for models that are in the early stages of refinement and model building or that only have very low-resolution data.

Not all server jobs complete successfully; currently, an average of 3% of submitted jobs stop prematurely. Limitations of the pipeline as well as problems with the input data may cause these stops. For instance, *PDB_REDO* cannot deal with unmerged reflections or with models derived from multi-model refinement at the moment. Examples of problems with the input data are atom names conflicting with the PDB standard or different residues with the same chain ID and residue number that are not each other’s alternates. In most cases an error message describing the problem and possible solutions is given when the optimization process is halted. Further development is needed to improve the feedback to the user, but we have already corrected many problems that have occurred in the server, thus continuously improving the *PDB_REDO* pipeline.

Any automated tool has the risk of becoming a black box, and the *PDB_REDO* server is no exception. To reduce this risk, many items in the output of the server (*e.g.* the validation metrics and the choice of the *B*-factor model) are hyperlinked to a ‘frequently asked questions’ web page for background information. The explicit comparison in the output of the input model and the final model and the visualization scripts for *Coot* will hopefully also encourage users to critically review the results.

## 7. Conclusions   

The *PDB_REDO* web server is a tool to optimize structure models by refinement and model rebuilding. Significant model changes are highlighted at the global (macromolecular entity) and at the local (residue) level. The use of *PDB_REDO* can lead to substantial model improvements in terms of fit to the experimental data and geometric quality, provided that the initial model is a reasonable representation of the experimental data.
